# Psychopharmacological Treatment, Intraocular Pressure and the Risk of Glaucoma: A Review of Literature

**DOI:** 10.3390/jcm10132947

**Published:** 2021-06-30

**Authors:** Adela Magdalena Ciobanu, Vlad Dionisie, Cristina Neagu, Otilia Maria Bolog, Sorin Riga, Ovidiu Popa-Velea

**Affiliations:** 1 Neuroscience Department, Discipline of Psychiatry, Faculty of Medicine, ‘Carol Davila’ University of Medicine and Pharmacy, 020021 Bucharest, Romania; 2 Department of Psychiatry, ‘Prof. Dr. Alexandru Obregia’ Clinical Hospital of Psychiatry, 041914 Bucharest, Romania; romcrys@yahoo.com; 3 Department of Psychiatry and Psychology, ‘Carol Davila’ University of Medicine and Pharmacy, 020021 Bucharest, Romania; 4 Service d’Ophtalmologie, Centre Hospitalier ‘Rene Dubos’, 95300 Pontoise, France; bologotilia@gmail.com; 5 Department of Stress Research and Prophylaxis, ‘Prof. Dr. Alexandru Obregia’ Clinical Hospital of Psychiatry, 041914 Bucharest, Romania; D_S_Riga@yahoo.com; 6 Romanian Academy of Medical Sciences, 927180 Bucharest, Romania; 7 Department of Medical Psychology, Faculty of Medicine, ‘Carol Davila’ University of Medicine and Pharmacy, 020021 Bucharest, Romania; ovidiu.popa-velea@umfcd.ro

**Keywords:** glaucoma, intraocular pressure, antidepressant, antipsychotic, benzodiazepine, topiramate, SSRI, SNRI

## Abstract

Through the years, the available psychopharmacological treatments have expanded with numerous new drugs. Besides weight gain, gastro-intestinal problems or Parkinson-like symptoms, ocular adverse effects of psychiatric drugs have been reported. These adverse effects are not common, but can be dangerous for the patient. This review summarises the current knowledge on the risk of raised intraocular pressure and glaucoma entailed by psychopharmacological treatment. Also, it provides updated data for clinicians involved in the treatment of patients with glaucoma or glaucoma risk factors. For this purpose, we performed an extensive literature search in the PubMed database using specific terms. Selective serotonin and noradrenaline reuptake inhibitors are the best evidenced as having no association with glaucoma. Antipsychotics, and especially first generation, seem to have no correlation with an increased intraocular pressure and therefore possibly with a risk of glaucoma, although a special attention should be paid when using ziprasidone. Tricyclic antidepressants, benzodiazepines and topiramate should be avoided in patients diagnosed with glaucoma or at risk. Clinicians should be aware of the possible psychotropic drug induced glaucoma and monitor at risk patients closely in order to prevent this condition. Irrespective of the psychopharmacological regimen taken into consideration, the glaucoma patient should be under the strict supervision of the ophthalmologist.

## 1. Introduction

Glaucoma represents a heterogeneous group of chronically progressive neurodegenerative bilateral diseases of the optic nerve, clinically characterized by optical neuropathy, resulting in retinal ganglion cell death, optic nerve head cupping, and associated specific loss of the visual field [1,2,3]. The aetiology of the disease is considered to be multifactorial [4], while the clinical picture can differ, with a substantial risk of associated blindness, especially in adults over the age of 60 [5].

Studies have shown that in Europe glaucoma occurs in 2.93% of people aged 40 to 80, reaching 10% at the age of over 90 [6]. Several types of glaucoma are described, which form a group of eye diseases and are the main cause of permanent blindness worldwide. [7,8]. Based on the mechanism by which aqueous outflow is impaired with respect to the anterior chamber configuration, the disease is typically divided into 2 basic subtypes: open angle and angle closure [9,10]. Clinical presentation of open-angle glaucoma (OAG) includes mainly chronic, slow and irreversible loss of peripheral vision, ultimately leading to blindness. Because of the gradual and insidious development, more than 50% of patients are unaware of their condition (known as ‘the sneak thief of sight’), especially due to the pattern of visual field loss that spares the central vision until advanced stages [9]; therefore, periodic ophthalmologic evaluation is important [11,12]. On the other hand, acute angle-closure glaucoma (AACG) represents a dramatic urgent event that is caused by the sudden increase in the intraocular pressure (IOP) due to occlusion of the trabecular meshwork by the peripheral iris (in predisposed eyes with a narrow iridocorneal angle), obstructing aqueous outflow, that can lead to a potential risk of rapid blinding. ACG patients usually present more acute symptoms such as hyperaemia, teary, and painful eyes, sudden blurring of vision, or halos around lights secondary to corneal oedema from a sudden rise in IOP. Increased IOP is responsible for additional symptoms such as headache, nausea, and vomiting [9]. It must also be kept in mind that a narrow angle can be asymptomatic in the absence of a predisposing factor for angle-closure (e.g., pupillary dilatation) or that there are cited cases of insidious angle-closure glaucoma, which tends to be more visually destructive subtypes [9].

Above all, we have to make a firm distinction between acute angle-closure and angle-closure glaucoma. The major difference between the two entities is the absence and presence of optic nerve damage and visual field defects, respectively, especially when a specific treatment is rapidly received by the patient. The notion of glaucoma is under discussion when an optic neuropathy with elements specific to glaucoma damage occurs.

The scientific literature identifies several local and systemic risk factors associated with the development and progression of glaucoma [13]. In the case of OAG the local risk factors are represented by IOP (the key modifiable factor), family history of primary OAG, intraocular anti-VEGF (vascular endothelial growth factor) therapy, decreased thickness of the central region of the cornea, pre-existing myopia (low–moderate and high), low intraocular vascular pressure, optic nerve pathology, visual field changes, disc haemorrhage, and pseudo-exfoliation. Systemic risk factors include cardiovascular diseases, diabetes mellitus, dyslipidaemia, cerebral stroke, steroid treatment, arterial hypertension, with old age and male gender acting as additional risks [8,10,14]. Concerning the rapid progression of glaucoma, the most important favouring factors are considered to be high IOP and cardiovascular diseases [15]. 

On the other hand, the risk factors for developing ACG mentioned in the literature are the following: age (62 years being the average age at presentation), gender (more commonly female), race (Asian descent), family history, hyperopic eyes, short eyes. Those at risk for ACG with packed angle configuration can develop an attack exacerbated by mydriasis either spontaneously (primary) or pharmacologically (secondary) [9,10].

Medication represents a distinct risk for glaucoma. Corticosteroids are the most common cause of open-angle glaucoma (OAG), but several non-steroidal anti-inflammatory agents can also lead to OAG [16]. Regarding ACG, the list of potentially risky drugs includes antidepressants, such as selective serotonin reuptake inhibitors (SSRIs), tricyclic antidepressants and monoamine oxidase inhibitors, antipsychotic drugs, antihistaminic medication, antiparkinsonian agents, anticonvulsants (e.g., topiramate), mydriatic agents, sympathomimetic drops, antispasmodic drugs, and botulinum toxin. 

Among the two types of glaucoma, ACG represents a pathology of interest concerning psychiatric treatment. Medications with anticholinergic effects could induce a precipitation of iridocorneal angle closure in patients with predisposition via mydriasis which is the primary pathogenic mechanism in the appearance of glaucoma in psychiatric patients [17,18] This rapid onset of the angle obstruction produces an imbalance between the production and drainage of aqueous humour in the anterior chamber, thus an accumulation of liquid and the increase of IOP. Other presumed involved mechanisms in the development of glaucoma are the anterior dislocation of the iris and lens or the inflammation of the ciliary body [7]. 

The challenges presented by glaucoma in psychiatry are bidirectional: on the one hand, patients with risk factor for angle-closure may develop clinical symptoms (eye pain, visual changes, headache) as a direct side effect of the psychiatric treatment. In case of patients with non-diagnosed OAG the raise in IOP can lead to severe aggravation of the disease, which can pass unnoticed until advanced stages [19]. On the other hand, glaucoma patients may exhibit a wide array of psychiatric symptoms, as a consequence of progression of vision loss, such as depression [20], anxiety [21] and insomnia [22].

Therefore, a minimal and specific screening before initiating a psychiatric treatment should be kept in mind, at least for the patients who have risk factors to develop OAG (IOP measure, fundus examination, +/− optic coherence tomography—OCT of the optic nerve head) or ACG (IOP measure, gonioscopy, +/− OCT of the optic nerve head) depending on the mechanism of action of the class of drugs used. Nevertheless, a good multidisciplinary collaboration between the psychiatrist and the ophthalmologist is strongly recommended, especially in complex cases.

Given the importance of glaucoma in influencing the choice of psychiatric drugs, we propose to review the main psychiatric therapeutic agents and their potential effects on glaucoma occurrence. Secondly, our aim is to provide a useful review of current data for clinicians facing dilemmas regarding the pharmacology treatment of psychiatric disorders in patients with glaucoma or glaucoma risk factors. 

## 2. Methodology

We conducted an extensive literature search in the PubMed database from 1977 until 2021. The search was performed during March-May 2021 using the following terms: ‘psychotropic medication’, ‘SSRI’, ‘citalopram’, ‘escitalopram’, ‘paroxetine’, ‘fluvoxamine’, ‘fluoxetine’, ‘sertraline’, ‘SNRI’, ‘duloxetine’, ‘venlafaxine’, ‘tricyclic antidepressants’, ‘clomipramine’, ‘amitriptyline’, ‘imipramine’, ‘doxepin’, ‘desipramine’, ‘nortriptyline’ ‘NDRI’, ‘bupropion’, ‘benzodiazepine’, ‘alprazolam’, ‘diazepam’ ‘antipsychotics’, ‘haloperidol’, ‘ziprasidone’, ’risperidone’, ‘olanzapine’, ‘quetiapine’, ‘clozapine’, ‘topiramate’ cross-referenced with ‘glaucoma’ and ‘intraocular pressure’. We selected only articles written in English and based on clinical reports. After review of title, keywords and abstract, we retrieved 128 articles. Following removal of duplicates, full text assessment and then screening of the remaining articles for relevant studies that could be included in our paper, we finally included 90 articles divided as it follows: SSRI-7, citalopram-2, escitalopram-2, paroxetine-5, fluvoxamine-1, sertraline-1, SNRI-2, duloxetine-2, venlafaxine-5, tricyclic antidepressants-1, clomipramine-1, amitriptyline-1, imipramine-1, bupropion-4, benzodiazepine-2, diazepam-1, antipsychotics-8, haloperidol-3, risperidone-1, topiramate-40.

## 3. Antidepressants

### 3.1. Selective Serotonin Reuptake Inhibitors (SSRIs) and Serotonin and Noradrenaline Reuptake Inhibitors (SNRIs)

SSRIs and SNRIs are currently the first line drugs for the treatment of depression according to international guidelines [23,24]. SSRIs and SNRIs are the most prescribed drugs for depression and have the best overall tolerability and safety among all antidepressants. Also, these drugs are indicated as first choice for the treatment of anxiety, post-traumatic and obsessive compulsive disorders [25,26,27].

**Figure 1:**
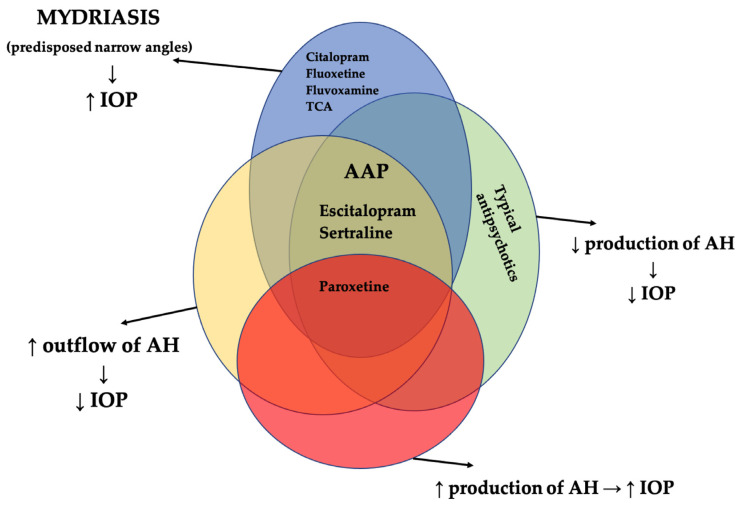
Schematic representation of the relationship between antidepressant and antipsychotic drugs and the various ocular side effects related to glaucoma pathogenic mechanisms. AAP, atypical antipsychotics; IOP, intraocular pressure; AH, aqueous humour; TCA, tricyclic antidepressants; ↑, increased; ↓, decreased.

Since the discovery in 1974 of the first member of SSRI class, namely fluoxetine, continuing with sertraline, paroxetine, fluvoxamine, citalopram and escitalopram, these drugs have revolutionised the pharmacological therapy of depression. SSRIs mechanism of action implies a selective blockage of the reuptake of serotonin in the synaptic gap, therefore increasing the availability of the neurotransmitter and normalising the function of synapses. Until now seven families of serotonin receptors (5HT1-5HT7) have been described as having a diffuse localisation, including eye structures. Experimental studies have determined that 5HT1A, 5HT2A/2C and 5HT7 are located in the iris-ciliary body complex. Stimulation of 5HT1A receptor reduces the IOP through the reduction of aqueous humour, while 5HT2A/2C receptors increase IOP by stimulation of the ciliary body blood flow, therefore they enhance the production of aqueous humour. 5HT7 receptors are responsible for mydriasis through the relaxation of the sphincter muscle and for rising IOP by increasing the production of aqueous humour [28,29]. In addition, mydriatic effects might appear due to their weak anticholinergic and noradrenergic actions [30]. These contrary possible effects of stimulating serotonin receptors have determined researchers to study the possible real relationship between glaucoma and SSRIs and SNRIs in order to shed some light on the field (Table 1, Figure 1).

The SNRI class has similar indications in psychiatry to SSRI, although they have been later on introduced, in the twentieth century. The SNRI class comprises venlafaxine (the first drug discovered from this class) and duloxetine. Their main mechanism is the inhibition of serotonin and norepinephrine reuptake, with weak dopamine transporter blockage. Noradrenaline is suggested to cause mydriasis and lid retraction through stimulation of α1 receptors; α2 inhibitory receptors from the ciliary epithelium can cause an increase in outflow facility of the aqueous humour while the blockage of these receptors by SNRI could reverse these effects leading to increased IOP [31]. The noradrenergic effect of SNRI is more dominant than the one of SSRI, suggesting a possible high risk of ACG. However, current data suggest that the systemic usage of SNRI could lead in long-term treatment to a decrease in the IOP. Another possible cause of mydriasis due to SNRI treatment would be the stimulation of serotoninergic receptors, mainly 5HT7, which in turn could lead to relaxation of the sphincter muscle [29,32].

#### 3.1.1. OAG

Zheng et al. (2018) documented a potential negative association between SSRIs with primary open angle glaucoma (POAG). More precisely, Zheng et al. (2018) showed that POAG patients treated with systemic medication and under SSRI therapy have a significant lower risk of developing POAG that would require a procedure (patients undergoing treatment with SSRI were at an averagely 30% lower risk for the development of OAG than non-SSRIs users). An approximately 30% reduced risk was also associated with the SNRI class, although less significantly. Also, it has been suggested that there is a dose-response relationship with lower odds of POAG with greater days of treatment [41].

These findings are similar to another cross-sectional study in which three groups of patients with open angle eyes were compared (patients receiving SSRI for 1 week to 6 months, longer than 6 months, or patients under no treatment). IOP was lower in patients under SSRI treatment for less 6 month or more than 6 months in comparison with controls, but the pupil diameter was higher in the abovementioned groups [30].

In contrast, a rise in IOP was documented in the case of a patient with chronic OAG during the initiation of treatment with venlafaxine (a SNRI). After the patient complained of headaches, the starting dose of 225 mg was reduced to 75 mg. No symptoms were reported after the dosage was lowered. Although asymptomatic at 3 months, the IOP increased and the retinal nerve suffered damages [42].

#### 3.1.2. ACG

Regarding ACG, in a large population-based study it was associated with a recent exposure to antidepressants in older adults [43], whereas long exposure to SSRIs did not influence the risk of ACG [44]. Another study conducted by Chen et al. (2017) concluded that individuals under SSRIs therapy had a greater risk of glaucoma (OAG, PACG, glaucoma state, glaucoma suspicion, other forms of glaucoma) incidence. Also, long-term use (>365 days) and/or high dosage were associated with a greater risk of developing glaucoma with an additive effect when both variables were included [37]. In a case-control study that included 1456 ACG patients, immediate SSRI users had 5.80 higher chances to develop ACG as compared to nonusers [45].

Conversely, a recent meta-analysis outlined that treatment with SSRIs was not associated with a higher risk of POAG or PACG and that IOP seemed to be lower in patients exposed to SSRIs. Also, Wang et al. (2018) concluded that pupillary diameter was higher in subjects under this type of antidepressant treatment [46].

Regarding the individual risk of each drug from the SSRIs class to cause glaucoma, current research is scarce and only a few case reports are available in the literature.

Citalopram-induced glaucoma (unilateral ACG) was reported in the literature in a case of drug overdose where the patient presented with blurred vision, pain and corneal oedema, in association with a high IOP. This patient was noted to have shallow anterior chambers in both eyes. After initial ophthalmologic treatment her IOP maintained normal without anti-glaucoma maintenance treatment [47]. Another case of citalopram induced bilateral symptomatic acute angle closure was reported in a patient with a history of 5 months of treatment with a normal dosage (the patient presented with blurred vision and headache) [48].

A case report of escitalopram-induced bilateral ACG described ciliochoroidal effusions after 4 weeks of treatment with a daily dose of 20 mg in a patient that presented with blurred vision. Ophthalmic examination revealed: high IOP, bilateral shallow anterior chamber, best corrected visual acuity was 20/40 bilaterally and a myopic shift of 4 dioptres over the current spectacle prescription. The condition was resistant to medical and surgical treatment but the patient recovered completely after escitalopram was discontinued [49]. Another article reported headache, blurred vision, vomiting, and photophobia (typical symptoms of AACG) in a patient that suddenly stopped escitalopram 1 month before the debut of symptoms. The risk factors identified in this case included hypertension (under control with beta-blockers) and escitalopram use for 1 year [50]. 

Patients under paroxetine therapy have reported ACG specific symptoms (i.e., loss of visual acuity or blurred vision) between 1 day and 4 months of treatment [51,52,53,54,55]. Interestingly, Sierra-Rodriguez et al. (2013) presented a case report of a unilateral visual loss due to chronic ACG under paroxetine treatment for 4 months. After discontinuation of paroxetine and laser iridotomy, the IOP normalised. Unfortunately, the patient resumed treatment on her own with consequent IOP rise despite patent iridotomies [53].

Regarding fluvoxamine, a patient with a previous history of narrow angle glaucoma (with no iridectomy) presented with daily headaches for 3 months and depressive symptoms (for anxiety the patient was taking lorazepam 2mg/day) and was prescribed fluvoxamine. After two months treatment the patient reported severe orbital pain and blurred vision (increased IOP and mydriasis). Despite specific therapy, IOP decreased only after the withdraw of the antidepressant [56].

Similar ACG symptoms were reported after three days of sertraline treatment, in a 64 year old Chinese woman with hypermetropia. It is worth to mention that Chinese ethnicity, old age, female gender and hypermetropia are risk factors for AACG [57]. 

Concerning SNRIs, the current literature describes only two and four ACG case reports involving duloxetine and venlafaxine, respectively.

The possible association of duloxetine with the appearance of ACG symptoms was reported in two female patients (46 and 81 years old, respectively). It is important to mention that the 81 years old patient was suffering from other comorbidities, hypermetropia and cataract [33,58].

Regarding venlafaxine, literature reports 4 possible cases of AACG after recent administration of this antidepressant, with the onset of symptoms ranging between 4 h and 10 days. In all reported cases, the patients were females of different age (between 35 and 70 years old) and had blurred vision as a common symptom [59,60,61,62]. 

Taking into account all the described current literature data, SSRIs and SNRIs have in general no association with either types of glaucoma or increased IOP. Moreover, it is worth emphasizing that long term treatment with SSRIs or SNRIs is associated with a decrease in the IOP, which suggests a possible protective effect of these drugs that needs further investigation. Of course, studies that assess the relationship between specific SSRIs or SNRIs and the risk of raised IOP and glaucoma are necessary in order to better characterise each drug regarding this possible side effect. The current case reports that describe a possible relationship between a specific SSRI or SNRI and ACG should warn the prescribers to closely monitor patients during treatment, especially the individuals with associated risk factors.

### 3.2. Bupropion

Bupropion is a noradrenaline and dopamine reuptake inhibitor (NDRI) and has been used since 1985 mainly as an antidepressant and more recently as adjuvant for smoking cessation. Bupropion is known to have anti-tumour necrosis factor (TNF) effects and a decreased activity on acetylcholine receptors that result in less anticholinergic side effects [63]. Studies hypothesised that IOP might be raised by TNF through increased caspase activity or mitochondrial dysfunction in the aqueous humour outflow channels. TNF synthesis is decreased by noradrenaline (β2 receptor) and dopamine (D1 receptor) activation [39,64]. All these effects led to the possible conclusion that bupropion could have some protective proprieties regarding IOP and glaucoma (Table 1).

A cross-sectional study that included patients over 40 years old investigated the relationship between self-reported glaucoma and self-reported bupropion use for at least 1 year. Masis et al. (2017) concluded that the usage of bupropion for longer than one year may be associated with a lower risk of self-reported glaucoma. Other covariates associated with high risk included Hispanic/Black ethnicity, increased age, cataract extraction, and diabetic neuropathy. One important limitation of this study is the lack of specificity in glaucoma type (ACG or OAG) [64]. 

#### 3.2.1. OAG

A cohort-type study regarding the risk of OAG coupled with bupropion treatment, reported a reduced hazard of developing this type of glaucoma. More precisely, the percentage of bupropion users that developed OAG was 1.8% and the percentage of non-users who developed OAG was 2.4%. Moreover, usage of this drug for 24–48 months has been associated with a 21% reduced chance of OAG [39]. 

#### 3.2.2. ACG

A case-control study conducted on patients under 50 years old reported bupropion treatment to be associated with an increased risk of ACG. No new prescriptions were issued afterwards, which could imply that predisposed eyes with narrow angles and pupillary dilation were not likely since iridotomy would have allowed continuation of treatment. Although the manufacturer’s information references the occurrence of ACG secondary to a pharmacological pupillary dilation, there is a possibility that choroidal effusion can occur [36]. 

Also, 2 weeks bupropion treatment (300 mg/day) was incriminated as a cause ACG in a 40 years old woman, with complains of blurred vision. Ultrasound bio microscopy revealed bilateral choroidal effusions that caused shallow angles [65].

### 3.3. Tricyclic Antidepressants (TCAs)

TCAs are the first generation of antidepressants and have been used in the psychopharmacological treatment of depression since around 1950. TCAs inhibit, through action on specific transporters, the reuptake of serotonin and noradrenaline in the synapse cleft. Also, TCAs block the postsynaptic histamine, acetylcholine and alpha-adrenergic receptors. Unfortunately, due to the cardiovascular and gastrointestinal serious adverse effects and their lethality in overdose quantities, TCAs have been replaced over time by SSRIs and SNRIs in the management of depression [66].

According current literature data, tricyclic antidepressants (clomipramine, amitriptyline, imipramine, doxepin, desipramine, nortriptyline etc.) are reported to be involved only in ACG and to precipitate AACG. The pupillary block via pupil dilatation that occurs during treatment with TCAs is attributed to the significant anticholinergic and serotonergic effects of these antidepressants [67]. The most frequent anticholinergic effects on the eye are mydriasis and cycloplegia, which in turn may cause the blockage of the trabecular meshwork. These effects result in blurred vision due to loss of accommodation and in precipitation of ACG [67,68,69,70] (Figure 1).

A relative large body of case reports that linked different TCAs treatment to the raise of IOP and glaucoma occurrence is present in the literature. Schlingemann et al. (1996) described the case of a 59-year-old woman with developed monocular vision loss, increased IOP and narrowed anterior chambers supposing due to treatment with clomipramine (75 mg/day) [71]. Lowe et al. (1966) also reported the cases of 4 patients on small dosage amitriptyline therapy that developed ACG [72]. In addition, Ritch et al. (1994) documented 4 cases of narrow angled patients who developed ACG related to imipramine treatment. Ritch et al. stated that uveal tract problems could be associated with TCA, mydriasis being often transient, without major consequences. Moreover, ACG can be promoted in susceptible patients (e.g., narrow angle individuals) [73].

All things considered, current data point out the risk of using drugs with potent anticholinergics proprieties, such as TCAs. The development of the new classes of antidepressants (i.e., SSRI and SNRI) provides important alternatives. In the case that TCAs must be used, the drugs with the less anticholinergic effects, such as desipramine and nortriptyline, should be taken into consideration [74]. No doubt, further cohort studies that assess the potential risk of angle closure glaucoma associated with TCAs are necessary in order to make conclusive recommendations.

## 4. Benzodiazepines (BZD)

Benzodiazepines are among the most commonly prescribed drug class in psychiatry and exhibit sedative, hypnotic, anxiolytic and muscle-relaxing properties by enhancing the effect of gamma aminobutyric acid (GABA). Due to this effect, benzodiazepines are incriminated to influence the sphincter pupillae and to determine the narrowing of iridocorneal angle [75]. Current literature documents only the relationship of BZD with ACG and AACG. 

Until recently, few cases have been reported about the association of AACG with BZD treatment. The conclusions were rather ambiguous, given the fact that other psychotropic agents have been concomitantly used during the time AACG was reported to occur [75]. Park et al. (2019) tried to reveal the clear relationship between benzodiazepine usage and the risk of glaucoma. In this population-based case-control study on elderly patients, who are more susceptible to the adverse effects to BZD, the authors demonstrated a significant correlation between immediate new use of BZD (within 7 days of AACG diagnosis) and the occurrence of AACG. Oppositely, no significant change in AACG incidence in the non-immediate new users was reported. In addition, there was no significant difference between short half-life (<24 h) vs. long half-life (>24 h) benzodiazepine agents [76]. These findings are similar to the ones of Kim et al. (2020), who outlined an association between BZD therapy and AACG in a cohort of 6709 patients [75]. In the study group the most frequent prescribed BZD were Diazepam and Alprazolam. These drugs were also associated with the highest risk of AACG occurrence [75]. However, a different study concluded that diazepam reduced the IOP and would actually be safe in procedures where lowered IOP is desirable [77].

Therefore, we conclude that benzodiazepines could precipitate ACG in predisposed eyes and clinicians should be aware of these possible side effects.

## 5. Antipsychotics

Antipsychotic drugs are the cornerstone in the management of schizophrenia. Other indications for this category include schizoaffective disorder, bipolar disorder, delusional disorder, severe agitation, delirium, or psychotic features of major depressive disorder. They can be divided in two categories: typical or first generation (Haloperidol and Chlorpromazine as most known) and atypical or second generation (clozapine, asenapine, olanzapine, quetiapine, paliperidone, risperidone, sertindole, ziprasidone, zotepine, and aripiprazole). Typical antipsychotics manage the symptoms of psychosis through the blockage of the dopaminergic postsynaptic receptors, with additional histaminergic, α1 adrenergic and cholinergic blockade. Atypical antipsychotics are serotonin and dopamine antagonists with affinities for serotonin (5HT1A, 5HT2C, 5HT6, 5HT7), dopamine (D1, D3, D4) receptors, but also histamine (H1), muscarinic (M1, M2, M3, M4, M5) and adrenergic (α1, α2) receptors [78]. Most AAPs have in common a more potent 5HT2A action than dopamine (D2) antagonism. They are also partial agonists of 5HT1A. All these features contribute to the low extrapyramidal effects. Some other actions include the inhibition of muscarinic receptors (anticholinergic activity). A possible explanation for the association between antipsychotic drugs and glaucoma might be the cholinergic receptor blockade (muscarinic) [34,79] (Table 1, Figure 1).

Unfortunately, current research reports only the possible effects of antipsychotics on IOP and does not scrutinise the relationship between these drugs and OAG or ACG. 

A cross-sectional study including 28 patients with schizophrenia showed that individuals under typical antipsychotics treatment had normal IOP [79]. This result is similar to the one reported by Reid et al. (1976). Moreover, Reid et al. (1976) study concluded that there was no IOP raise despite high dosage of typical antipsychotics [80]. Actually, not only haloperidol was found to lower IOP in glaucomatous eyes, but also it was proposed as a possible treatment for glaucoma [81,82,83].

Atypical antipsychotics (AAP) have cardiovascular side effects through the vasoconstriction determined by α1 adrenoreceptors blockage. Risperidone, clozapine, iloperidone and quetiapine may lead to hypotension via this mechanism [34]. Animal studies showed instilled APP into the eye determined a significant reduction of IOP observed after 1 h. Also, reduction of blood pressure occurred within 10 min after administration [84].

Reactive oxygen species (ROS) production is involved in the pathogenic mechanism of glaucoma. Risperidone was demonstrated to have the ability to decrease oxidative stress (OS) in schizophrenic patients by controlling the inflammatory response [85]. Other AAP that have been shown to decrease OS are clozapine and olanzapine [86]. However, the relationship between AAP, OS and glaucoma has been incompletely investigated. Therefore, future studies are necessary to elucidate this possible mechanism.

Several studies described that IOP elevation may lead to the inhibition of brain-derived-neurotrophic factor (BDNF) which could have in turn a contributing effect to the visual loss. Risperidone and clozapine have been found to increase levels of BDNF [87,88,89].

There is some evidence that AAPs could enhance glaucoma through anti-muscarinic action. For example, clozapine and olanzapine have high affinity for muscarinic receptors (inhibition) and anticholinergic activity, which could possibly exacerbate glaucoma [90]). Thus, the actions of AAP by downregulating OS and neurotrophins may be unbalanced because of their anti- muscarinic receptor action. This observation could explain that, in general, AAPs are not associated with a glaucoma risk [66].

A cross-sectional analysis of 28 patients with schizophrenia and under antipsychotic therapy (4 on typical antipsychotics, 16 on AAP, and 8 on both types) provided interesting conclusions. More precisely, a raise of IOP has been found only in patients under AAP therapy, particularly all on ziprasidone. Ziprasidone is known to exert a potent serotoninergic (5HT2A) and dopaminergic (D2) affinity [79].

In conclusion, clinicians should be aware that second generation antipsychotics could have some implication in the variations of IOP, therefore a special attention should be paid to patients at risk and also when prescribing ziprasidone. Increased IOP could have no clinical significance in certain situations (e.g., minor increase or lower basal IOP) or could lead to the development or progression of glaucoma (OAG or ACG) depending on the characteristics of the patient. Typical antipsychotics are suggested to be safer in relation to a possible rise of IOP.

## 6. Topiramate

Topiramate is a sulpha-derivative monosaccharide and it is commonly prescribed for treatment of epilepsy and migraine prevention. Off label indications include eating disorders, obesity and tobacco dependence. Regarding psychiatric recommendations, the current literature describes the benefits of topiramate in weight gain prevention and metabolic dysfunction in schizophrenic patients as a result of treatment with certain antipsychotics (i.e., olanzapine, clozapine) [91].

Topiramate’s mechanism of action involves inhibition of carbonic anhydrase, calcium channels, and glutamate receptors, as well as blockage of the sodium channels and stimulation of gamma-aminobutyric acid receptors. Topiramate is suggested to cause or worsen glaucoma due to an acute hypersensitivity reaction and alteration of osmotic status. The mechanism is suggested to involve ciliochoroidal effusion which leads to anterior rotation of the swelled ciliary body with anterior shifting of lens-iris diaphragm and consequently shallowing of the anterior chamber and narrowing of the angle [92,93]. All these changes may also occur in normal eyes. Given this fact, clinicians should be more vigilant and it is advisable to adopt a watchful waiting approach for all patients treated with topiramate.

In a retrospective study, Ho JD et al. (2013) reported a greater risk of developing ACG after topiramate therapy in the first month of treatment (hazard ratio 7.41 times higher than control subjects) [93]. Other data showed an increased risk of ACG, myopia, suprachoroidal effusion, and abnormal vision, all reversible with the discontinuation of treatment [94,95].

Numerous case reports found in the current literature described the association of topiramate treatment with the development of ACG. In 38 reported cases [96,97,98,99,100,101,102,103,104,105,106,107,108,109,110,111,112,113,114,115,116,117,118,119,120,121,122,123,124,125,126,127,128,129,130], patients under topiramate treatment developed ACG after a short time from the treatment initiation (the majority between 1 day and 14 days), most of them being adult women (27 women / 38 patients) with an age ranging from 23 to 59 years. Table 2 presents a summary of current topiramate induced acute angle closure or AACG case reports (Table 2).

## 7. Conclusions

Based on the presented data, clinicians should be aware of the glaucoma-related risk-benefit profile of psychotropic medication and tailor their recommendations. The selective serotonin and noradrenaline reuptake inhibitors class is the medication group with the most solid results regarding a minimal possible risk of glaucoma. More precisely, SSRI and SNRI treatment seems to have even a protective role regarding OAG and no effect in relationship with ACG. Therefore, practitioners could use these drugs safely since there is no risk of a glaucoma induced effect. On the other hand, tricyclic antidepressants should be avoided in patients at risk to develop angle closure glaucoma or in angle closure glaucoma diagnosed individuals due to their strong anticholinergic and antimuscarinic proprieties. Regarding all antipsychotics, there is an important gap in the knowledge of their relationship with the risk of glaucoma. Based on the herein reviewed data, first generation antipsychotics do not seem to affect the intraocular pressure, but for the second generation antipsychotics, and especially ziprasidone, further studies are needed in order to bring some light to the current data. Also, topiramate is another drug that we advise not be used in the treatment of patients with possible risk factors or diagnosed with angle closure glaucoma, since current data point to an increased risk of trabecular obstruction and consequently a raise in intraocular pressure. Benzodiazepines should be prescribed carefully, especially in older patients. Whether or not it is identified as a contraindication, physicians should be aware of the possibility of psychotropic drug-induced glaucoma, especially angle closure type, and if the suspicion of glaucoma arises, ophthalmological assessment is recommended. Early recognition of this possible side effect and discontinuation of the drug in question are measures that should be immediately employed by the psychiatrist concomitantly with referring the patient to an ophthalmologist for a thorough evaluation. Due to the vast psychotropic medication and possible mechanisms and their interactions, future studies are needed to fill the literature gaps and enrich current knowledge on this subject.

## Figures and Tables

**Table 1:** Mechanisms of ction of psychopharmacological drugs and their possible effect on intraocular pressure, pupil size and glaucoma risk.

Effector	Receptors	Location	Drugs	Possible Induced Effect	Effect on IOP
Adrenaline	𝛼1	Iris dilator muscle	Citalopram Escitalopram Paroxetine Fluvoxamine Fluoxetine Sertraline Ziprasidone Other atypical antipsychotics	Mydriasis (Hypertension, lid retraction) [33,34,35]	-
	𝛼2	Ciliary epithelium	Escitalopram, Paroxetine, Sertraline Atypical antipsychotics	Increased outflow of aqueous humour	↓IOP [33,35]
	𝛽2	Ciliary epithelium	Paroxetine	Increase production of aqueous humour	↑IOP [36]
Serotonin	5HT7	sphincter of the pupil, iris ciliary body	Paroxetine, Ziprasidone	Mydriasis	↑IOP [28,33,37]
	5HT1A	Iris-ciliary body	Escitalopram, Paroxetine, Fluvoxamine, Sertraline, Ziprasidone Other atypical antipsychotics	-	↓IOP [28,33,34,35,37]
	5HT2A	Iris-ciliary body	Escitalopram Paroxetine, Fluoxetine Sertraline, Venlafaxine, Ziprasidone Other atypical antipsychotics	-	↓IOP [28,33,37]
	5HT2C	Iris-ciliary body	Citalopram Escitalopram, Fluvoxamine Fluoxetine, Paroxetine, Sertraline, Venlafaxine Ziprasidone, Other atypical antipsychotics	-	↑IOP [28,33,35,37]
Dopamine	DA1	The ciliary body, trabecular meshwork, and uveoscleral tissue	Paroxetine	Increased production of aqueous humour	↑IOP [33,35,38]
	DA2	Anterior segment	Escitalopram Paroxetine, Sertraline, Ziprasidone Typical and other atypical antipsychotics	Suppression of the production of aqueous humour	↓IOP [33,35,38]
Acetylcholine (miotic effect)	Muscarinic (Blockade)	Smooth muscle around the pupil	Citalopram, Paroxetine Escitalopram, Fluoxetine. Sertraline Tricyclic antidepressants Typical and atypical antipsychotics	Mydriasis	↑IOP [36]
TNF	TNF-R1	Aqueous humour outflow channels	Bupropion	Increased caspase activity, mitochondrial dysfunction	↑IOP [39]
Sulpha based drugs	-	-	Topiramate	Allergic reaction (myopia, swelling of the ciliary body, forward displacement of the lens-iris diaphragm)	↑IOP [40]

↑, increased; ↓, decreased; IOP, intraocular pressure; 5HT, 5-hydroxytryptamine (serotonin); DA, dopamine; TNF, tumour necrosis factor; R, receptor.

**Table 2:** A summary of currently reported topiramate-induced acute (primary) angle-closure or acute (primary) angle-closure glaucoma cases (in adult patients).

Case Report	Patient’s Gender, Age and Other Comorbidities	Onset after Drug Initiation
Alzendi et al. (2020) [96]	Female, 24 yo, migraines	13 days
Agarwal et al. (2019) [97]	Female, 25 yo, morbid obesity, obstructive sleep apnoea	11 days
Mahendradas et al. (2018) [98]	Female, 36 yo, hypothyroidism	5 days
Sierra-Rodríguez et al. (2018) [99]	Female, 29 yo, epilepsy	9 days
Lan et al. (2017) [100]	Female, 43 yo, arrhythmia	4 weeks
Czyz et al. (2014) [101]	Female, 40 yo, arterial hypertension, degenerative disk disease, fibromyalgia, migraines, chronic obstructive pulmonary disease	262 days
Pikkel et al. (2014) [102]	Male, 54 yo	7 days
Katsimpris et al. (2014) [103]	Female, 36 yo, migraines	14 days
Quagliato et al. (2013) [104]	Female, 55 yo, migraines, spasmodic torticollis, essential tremor	7days
Caglar et al. (2012) [105]	Female, 36 yo, migraine	1 day
Cole et al. (2012) [106]	Female, 56 yo, depression treated with venlafaxine	2 days
van Issum et al. (2011) [107]	Male, 34 yo, epilepsy	14 days
Willett et al. (2011) [108]	Male, 39 yo, arterial hypertension, migraines	7 days
Natesh et al. (2010) [109]	Male, 23 yo	5 days
Acharya et al. (2010) [110]	Male, 49 yo	14 days
Spaccapelo et al. (2009) [111]	Male, 34 yo, anxious-depressive syndrome treated with citalopram	7 days
Sbeity et al. (2009) [112]	Female, 59 yo, keratomileusis surgery, myopia	11 days
Chalam et al. (2008) [113]	Female, 34 yo, arterial hypertension, hypothyroidism	7 days
Boonyaleephan et al. (2008) [114]	Female, 23 yo	7 days
Aminlari et al. (2008) [115]	Female, 48 yo, bipolar disorder, depression, hypothyroidism, chronic pain	14 days
Aminlari et al. (2008)	Male, 53 yo, cluster headaches, hyperlipidaemia	6 weeks
Singh et al. (2007) [116]	Female, 33 yo, headaches	7 days
Parikh et al. (2007) [117]	Male, 51 yo, epilepsy	14 days
Viet et al. (2006) [118]	Male, 57 yo, bipolar disorder	7 days
Sachi et al. (2006) [119]	Female, 33 yo, migraines	3 weeks
Rhee et al. (2006) [120]	Female, 35 yo, migraines	2 months
Levy et al. (2006) [121]	Female, 35 yo, depression	7 days
Desai et al. (2006) [122]	Female, 36 yo, migraines	10 days
Mansoor et al. (2005) [123]	Female, 51 yo, surgery for hypermetropia, migraines	7 days
Craig et al. (2004) [124]	Female, 25 yo, epilepsy, depression treated with Venlafaxine	7 days
Boentert et al. (2003) [125]	Female, 23 yo, congenital hydrocephalus, Arnold-Chiari formation I, partial atrophy of the right optic nerve, astigmatism, vertical strabismus.	6 days
Medeiros et al. (2003) [126]	Male, 44 yo	5 days
Medeiros et al. (2003) [126]	Female, 42 yo, myopia	10 days
Chen et al. (2003) [127]	Female, 42 yo, hypertension, seizures	2.5 weeks
Banta et al. (2001) [128]	Male, 51 yo, bipolar disorder	14 days
Sankar et al. (2001) [129]	Female, 34 yo, depression	14 days
Sankar et al. (2001) [129]	Female, 53 yo, depression treated with venlafaxine, high cholesterol.	10 days
Rhee et al. (2001) [130]	Female, 43 yo, depression treated with paroxetine	1 day

yo, years old.

## Data Availability

All data relevant to this paper are included in the article.
